# Dynamic contrast in dual-beam optical coherence tomography imaging of human skin

**DOI:** 10.1364/BOE.570414

**Published:** 2025-11-04

**Authors:** Michael Pircher, Elisabeth Brunner, Ionela-Mariana Nagelreiter, Christopher Kremslehner, Wenting Hu, Bahar Golabi, Christian Freystätter, Florian Gruber

**Affiliations:** 1 Center for Medical Physics and Biomedical Engineering, Medical University of Vienna, Waehringer Guertel 18-20, A-1090 Wien, Austria; 2 Department of Dermatology, Medical University of Vienna, Waehringer Guertel 18-20, A-1090 Wien, Austria; 3 Center for Brain Research, Medical University of Vienna, Waehringer Guertel 18-20, A-1090 Wien, Austria; 4 Department of Dermatology, Hangzhou Third People’s Hospital, 38 Xihu Road, Hangzhou, Zhejiang, 310009, China; 5 Department of Plastic, Reconstructive and Aesthetic Surgery, Medical University of Vienna, Waehringer Guertel 18-20, A-1090 Wien, Austria

## Abstract

Imaging of metabolic and cellular activity in living tissue is of high interest and represents a prerequisite for an understanding of environmentally induced tissue changes. In this work, we present a dual-beam optical coherence tomography (OCT) instrument that is used to investigate dynamic processes of human skin samples with two different transverse resolutions. The amplitude and the phase of the OCT signal in both channels are exploited to assess these processes. The temporal evolution of dynamic processes in living skin tissue is analyzed and used to provide increased image contrast. This allows for a clear separation between the stratum corneum, the living epidermis, and the dermis that is otherwise not visible in standard OCT images of the system. Thereby, better dynamic contrast was observed using the higher resolution images. The reproducibility of the method is tested, and the imaging results are compared with data from fixated skin samples (where no dynamics were found within the living epidermis) and histology.

## 1. Introduction

The skin, one of the largest organs of the human body, plays a key role in the protection from environmental influences. Aging processes [1], external insults and a large variety of diseases alter the normal function of the skin leading to a loss of its functionality as a physical and biological barrier and as an important organ for thermoregulation and the immune system. Although samples can in principle be easily retrieved and examined in detail by microscopic techniques, many aspects are preferably investigated *in situ* and *in vivo* as this allows observation of damage- or disease induced developments over time. Routinely, the skin is examined by dermatologists visually and with the aid of dermatoscopes in order to provide a magnified view of areas of interest. In addition, a multitude of different optical imaging techniques are meanwhile available including confocal microscopy [2,3], multi-photon imaging [4,5], near infrared spectroscopy [6] and optical coherence tomography (OCT) [7–10]. Alterations in squamous and basal cells can be indicative for the development of skin cancer and are frequently targeted with these imaging technologies [11].

One limitation of sole structural imaging lies in poor contrast (when imaged without cellular resolution) [12] and the inability to provide information on the functional status of tissue [13]. The latter is of specific interest as this relates to the metabolic activity of tissue. In OCT it has been shown that this can be overcome in living tissue by utilizing motion of subcellular components which results in fluctuations of the OCT signal [14]. Stunning images have been obtained by applying this technique to cell cultures which allowed for an assessment of normal cellular activity as well as cell death [15–18]. Recently, an overview of this technology has been presented [19]. While this earlier work relied on time domain full field OCT, the technique was soon extended to scanning Fourier Domain OCT systems and to a larger field of view [20–24]. Using this technology, additional contrast was found for example in excised tissue such as mouse trachea [25], human esophagus tissue [20], mouse liver tissue [26], human inner ear tissue [27] or in generated organoids [23,24]. A recent review article summarizes the various applications of this technique [28].

In this work we further extend the concept of dynamic OCT imaging by introducing a dual beam setup that allows for simultaneous imaging of tissue with two different transverse resolutions. The resolutions are chosen as a compromise between standard OCT skin imaging (7-15 µm resolution) [9] and cellular resolution imaging (1 µm resolution) [29] which results in a reasonable depth of focus for Fourier Domain OCT while providing high transverse resolution for dynamic OCT imaging. The instrument is used to investigate the dynamic contrast in living and fixated human skin samples. Thereby, the temporal characteristics of the amplitude as well as the phase of the OCT signal are analysed. The difference of dynamic image contrast caused by the differing transverse resolution of the OCT system is analysed and the reproducibility of the technique is outlined. In the living samples, increased dynamic contrast is observed from the living epidermis and the stratum corneum while in the fixated sample, no dynamic contrast is observed from the living epidermis. Our results indicate that imaging with higher resolution results in better visibility of dynamic contrast.

## 2. Method

### 2.1. Dual-beam swept source OCT setup

**Fig. 1.**
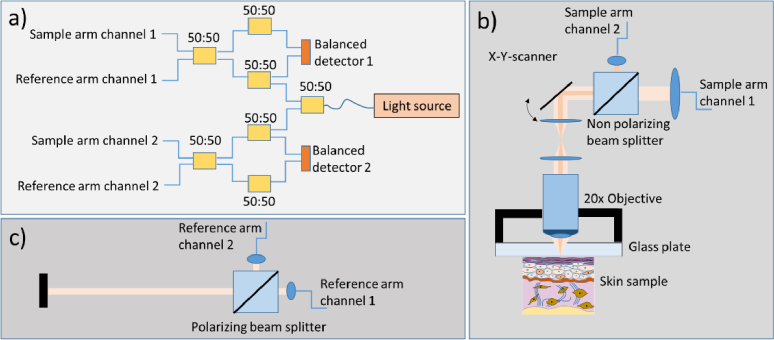
Scheme of the experimental setup. a) Two channel fiber based OCT interferometer using symmetric balanced detection. (For clarity the polarization paddles at the fibers of the sample and reference arms, respectively are omitted). b) Layout of the OCT sample arm depicting the beam path of the two OCT imaging beams. The different fiber collimators produce a different beam width and subsequently a different lateral resolution for the channels 1 and 2, respectively. c) Layout of the OCT reference arm depicting similar fiber collimators for both channels.

A scheme of the experimental setup is given in Fig. 1. We used the Axsun Technologies Inc. 1060 Swept Laser Engine as light source for the OCT system. The swept source laser is operated at a central wavelength of 1045 nm with a sweep range of 100 nm. The sweep or A-scan rate of the system is 100 kHz. The OCT interferometer is fiber based with a configuration as outlined in Fig. 1(a)). The first 50:50 fiber based beam splitter (FOBS-22P-1111-6/125-SSSS-980/1100-50/50-40-3A3A3A3A-1-2, OZ Optics Ltd.) splits the light of the light source into two beam paths of the OCT channels 1 and 2, respectively. The light in each OCT channel traverses another 50:50 fiber based beam splitter (identical to the first one) and the main OCT interferometer beam splitter that diverts 50% of the light into the sample and reference arms, respectively. The light returning from both arms is recombined by the main fiber based beam splitter. The light from both interferometer exits, each traverses a 50:50 fiber beam splitter before being detected by a balanced detector (PDB 460C, Thorlabs Inc.) and sampled by a dual channel digitizer (ATS9350, AlazarTech Inc.) The light for each OCT channel in the sample arm is collimated using two different lenses (f = 6.2 mm and f = 4 mm) that result in two different beam diameter for channels 1 and 2, respectively. A non-polarizing beam splitter is used to combine both beams co-linearly. The light is then directed to the x-y scanning unit and a telescope consisting of two lenses (each 50 mm focal length) images the pivot point of the scanner onto a 20x objective. The objective is connected to a glass plate that ensures stabilized focus within tissue (cf. Figure 1(b)). In the reference arm, the light from both OCT channels is collimated using identical collimators (cf. Figure 1(c)). A polarizing beam splitter combines the light co-linearly. The light is then back-reflected by a mirror and split into the components of each channel by the beam splitter. The use of a polarizing beam splitter minimizes cross-coupling of the reference arm light into the beam path of the other OCT channel. To ensure matching of the polarization state of the light returning from reference and sample arm, the fiber that is attached to the beam splitter before the sample arm collimator is coiled within a polarization control paddle. To match the reference and sample arm lengths between the two channels the collimators of channel 2 are each mounted on a translation stage. The power on the sample was measured to be 1.2 mW for channel 1 and 0.2 mW for channel 2, respectively. This results in a theoretical sensitivity of 99 dB for channel 1 and 92 dB for channel 2.

### 2.2. Scanning protocols and dynamic OCT evaluation

The system is operated with 512 A-scans per B-scan resulting in a B-scan rate of ∼195 Hz. Varying scanning protocols are used. For testing of the dynamic contrast and for visualization of metabolic activity using the different OCT components (amplitudes and phases of channels 1 and 2), we recorded 512 B-scans at the same location over a total duration of ∼2.7 seconds. The lateral extension of a B-scan is 750 µm. The depth extension of the system is 6 mm (in air) with a pixel spacing of 5.9 µm. To reduce the amount of data, only 300 pixel (equivalent to 1.77 mm in depth in air or 1.24 mm in skin tissue assuming a refractive index of 1.43) of the entire 1024 pixels are used for further processing. For 3 dimensional evaluation, we recorded 5 volumes scans, one after another, each consisting of 512 B-scans (same lateral spacing in the slow scanning direction as for the fast scanning direction) within 4 minutes. The recorded spectral data is corrected for dispersion (same parameter for both channels) by adding a quadratic phase term to the data before Fourier transformation [30]. Image correction (such that the surface of the glass plate is horizontally oriented in the images) is performed by adding a linear phase term depending on the lateral A-scan location to the spectral data before Fourier transformation which allows for image correction with sub-pixel accuracy [31]. This procedure is extended to a 3D volume scan such that a horizontal arrangement of the glass plate is achieved for the fast and the slow scanning axis of the volume, respectively.

The resulting amplitude data of a 2D scan is analyzed over time for each pixel by calculating either the standard deviation along time (512 time points in total) or by a fast Fourier transformation analysis (FFTa). The FFTa consists of subtraction of the mean amplitude (over time) and two times zero padding that is followed by fast Fourier transformation (FFT). After FFT, the information in 3 different frequency regions was extracted by summing of the corresponding values within these regions and by dividing by the number of values that each frequency region contains. The frequency regions were empirically found by averaging of the FFT of all image pixel that contain a signal above noise floor and by determining the present frequency peaks. We found that regions that spanned the range between <1 Hz, 1-10 Hz and 70-80 Hz provided the best image contrast for our skin samples. A false color scale image was then produced by assigning the colors blue, green and red for the logarithmic amplitude contained in these frequency bands.

For a phase analysis, each phase value within tissue is referenced to the surface phase value of the glass plate. This ensures that phase fluctuations that are caused for example by interferometric jitter or by jitter between the two channels are eliminated. In a next step, the mean phase value (for each pixel over time) is subtracted before applying 2x zero padding and the FFT. In contrast to the analysis of the amplitude fluctuations, the resulting Fourier amplitude in each frequency band is normalized by the sum of the Fourier amplitude that is contained in the entire frequency range. This step is essential in order to suppress pixels in the resulting images originating from regions with low signal or containing only noise. The phase based evaluation does not consider the amplitude information of the OCT signal but areas containing noise will have random phase values that result after FFT in a high amplitude over the entire frequency range. Similar to the evaluation of the OCT amplitude, the average amplitude within the 3 different frequency bands is extracted and the corresponding values are converted to a false color scale image.

**Fig. 2.**
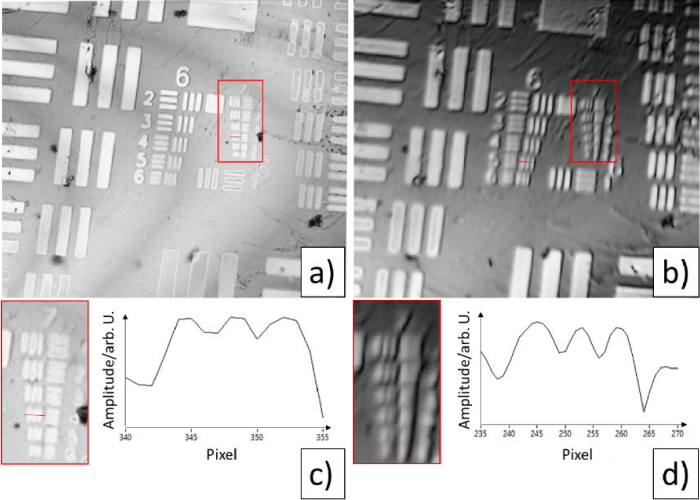
En-face images of a 1951 USAF resolution test target. a) Channel 1, b) channel 2. The red rectangles mark the regions of interest showing group 7 of the target and that are displayed enlarged below each figure. c) Line profile of channel 1 of element 7/4 (position indicated by the red line in a)). d) Line profile of channel 2 of element 6/5 (position indicated by the red line in b)).

We estimated the transverse resolution by determining the smallest resolvable element on the resolution test target (smallest element with a line profile that allows for a separation between the three bars). As can be seen in Fig. 2, the smallest resolvable element for channel 1 is group 7, element 4, while for channel 2 it is group 6, element 5. This translates to a lateral resolution of channel 1 and 2 of 2.76 µm and 4.92 µm, respectively.

### 2.3. Sample preparation and histological imaging

Skin samples were obtained from the Department of Dermatology. The skin samples used for this study were gained from plastic surgery procedures (abdominoplasty), the collection was approved by the Ethics Committee of the Medical University of Vienna (EK. Nr. 1169/2021) and written informed consent was obtained from all subjects. The use of these skin samples has been approved by the local ethics committee. After harvesting the tissue, small (∼1 cm in diameter) samples were extracted from the tissue. These were then stored in a nutrition solution and at body temperature in an incubator up to 24 hours before being imaged by the OCT. Fixation of tissue was performed by storing the sample in formalin for at least 24 hours. For histological staining the skin biopsies from the same subject but adjacent region of the OCT samples were similarly fixed in formalin and embedded in paraffin. The tissue block was cut into 4 µm sections and these were transferred onto glass slides. Then, slides were de-paraffinized in xylene and rehydrated in alcohol gradient and then stained with hematoxylin (Merck, Rahway, NJ, USA) for 10 min at room temperature, washed with tap water, and counterstained with eosin (Merck) for 5 min and imaged upon mounting and cover glass application. Imaging is then performed using a standard microscope (Olympus UC90).

## 3. Results

**Fig. 3.**
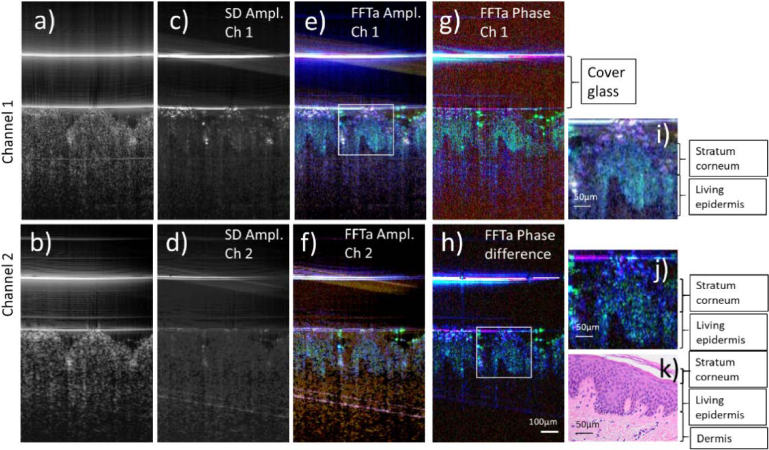
Representative images of a living human skin sample using the dual beam setup. a) Averaged OCT intensity B-scan of channel 1, b) averaged OCT intensity B-scan of channel 2 (both on a logarithmic scale and averaged over the full time lapse). c) and d) Images showing dynamic contrast calculated via standard deviation for channels 1 and 2, respectively. e) False color image retrieved from amplitude fluctuations in channel 1 indicating dynamics in different frequency bands. f) False color image retrieved from amplitude fluctuations in channel 2, g) False color image retrieved from phase fluctuations in channel 1. h) False color image retrieved from fluctuations in the phase difference between channels 1 and 2, respectively. i) 2x enlargement of the region of interest indicated by a white square in e). j) 2x enlargement of the region of interest indicated by a white square in h). (Color scale: < 1 Hz: blue, 1-10 Hz green, 70-80 Hz: red). OCT data retrieved from a total of 512 B-scans. k) Histology image of an adjacent skin region of the same sample.

Figure 3 shows imaging results of a living human skin sample obtained with the dual beam OCT system. Both averaged intensity images (cf. Figure 3(a) and (b)) show poor contrast between the skin layers stratum corneum, living epidermis and dermis. The dynamic image generated from channel 1 by calculating the standard deviation (SD) between all 512 B-scans (cf. Figure 3(c)) shows amplitude fluctuations caused by cellular or metabolic activity mainly at the living epidermis and the stratum corneum. Interestingly, this contrast is hardly visible in the SD image of channel 2. This indicates that the transverse resolution strongly influences the visibility of the dynamic OCT signal and therefore the ability to detect metabolic activity. A frequency analysis of the amplitude fluctuations reveals a strong metabolic activity in the 1-10 Hz frequency range (indicated in green) from the living epidermis and in a different frequency range from the stratum corneum (indicated in white and in violet) (cf. Figure 3(e)).

A similar analysis performed on the data from channel 2 reveals only increased activity from the living epidermis. Figure 3 g) shows a frequency analysis that was performed on the phase fluctuations in channel 1. A similar contrast was obtained as from the amplitude fluctuations. This indicates that the time scale of the fluctuations is similar for both, the amplitude and the phase of the OCT signal. Figure 3 h) shows a frequency analysis that was performed on the phase difference between channels 1 and 2, respectively. Again, a similar contrast within the image is achieved demonstrating the feasibility of the dual channel system to detect cellular or metabolic activity in skin tissue.

**Fig. 4.**
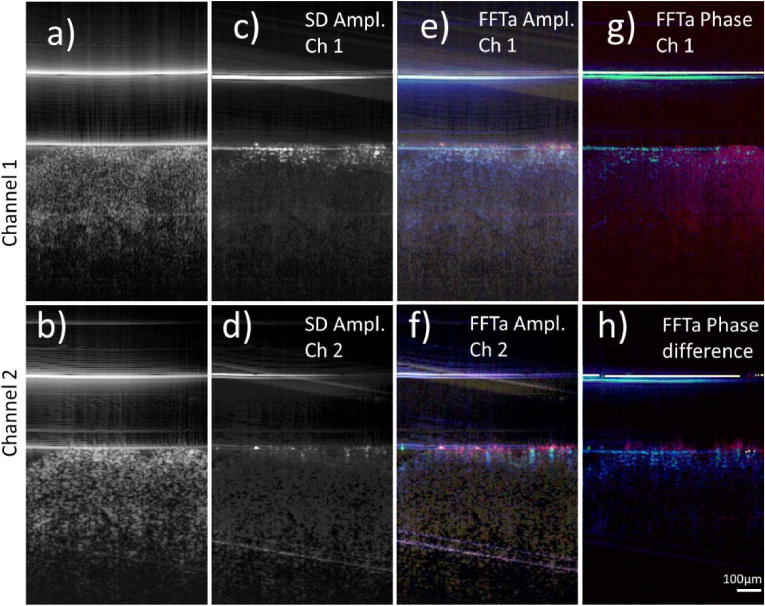
Representative images of a fixated human skin sample using the dual beam setup. a) Averaged OCT intensity B-scan of channel 1, b) Averaged OCT intensity B-scan of channel 2 (both on a logarithmic scale). c) and d) Images showing dynamic contrast calculated via standard deviation for channels 1 and 2, respectively. e) False color image retrieved from amplitude fluctuations in channel 1 indicating dynamics at different frequency ranges. f) False color image retrieved from amplitude fluctuations in channel 2, g) False color image retrieved from phase fluctuations in channel 1. h) False color image retrieved from fluctuations in the phase difference between channels 1 and 2, respectively (Color scale: < 1 Hz: blue, 1-10 Hz green, 70-80 Hz: red). OCT data retrieved from a total of 512 B-scans.

Figure 4 shows image results obtained from a fixated skin sample of the same subject and the same region. No activity from the living epidermis is detectable in all the different approaches for assessing temporal fluctuations. Solely the stratum corneum shows fluctuations in the amplitude and phase of the OCT signal. We attribute these fluctuations to fluid exchange processes between the stratum corneum and the storage liquid that is located between the glass plate and the tissue.

**Fig. 5.**
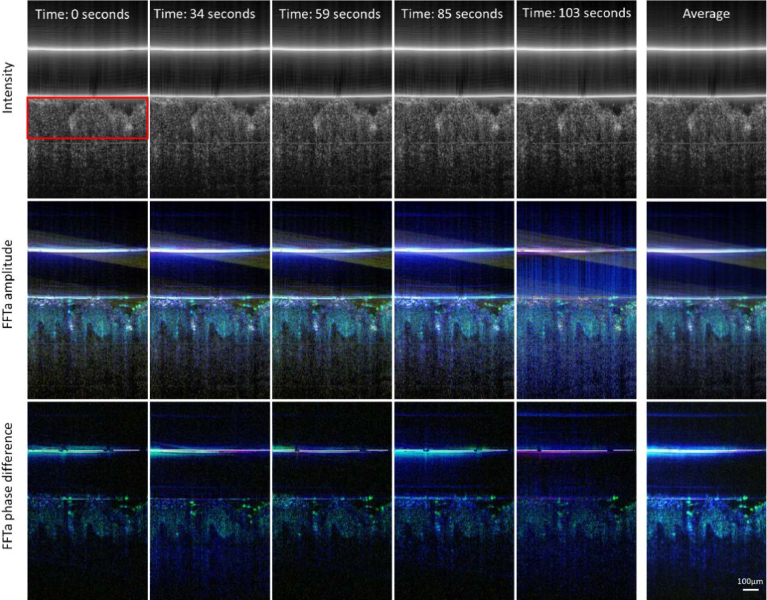
Representative images of a living human skin sample using the dual beam setup at different time points to test the reproducibility of dynamic image contrast. Top row: Intensity B-scan image of channel 1 (the red rectangle indicates the region of interest that was used for a quantification of the reproducibility), middle row: false color image retrieved from a frequency analysis of amplitude fluctuations in channel 1, bottom row: false color image retrieved from a frequency analysis of fluctuations in the phase difference between channels 1 and 2, respectively. The right hand column shows averaged images over all time points. (Color scale: < 1 Hz: blue, 1-10 Hz green, 70-80 Hz: red). OCT data retrieved from a total of 512 B-scans.

To test the reproducibility of the method, we recorded several data sets over a total time period of ∼100 seconds. The result is displayed in Fig. 5 and shows good qualitative reproducibility of the images between the recordings. The averaged data (right hand column) shows images with further improved contrast and quality. The artifact in the FFT analysis (vertical blue stripes) arises from trigger issues of the light source at some A-scans within the data that consequently results in amplitude fluctuations at lateral locations of the A-scans where these trigger issues occur. The cause of these trigger issues is unknown and were randomly observed in few of the data sets that we have recorded. Notably, is the removal of this artifact in the FFT analysis of the phase difference between the two channels because this trigger issue affects both channels.

To quantify the reproducibility we calculated the average and standard deviation of 4 out of the 5 images of the data recording. The last data set was omitted because of the already mentioned occasional trigger issues of the system. The result was averaged another time, now over a region of interest that is indicated by a red rectangle in the first frame of Fig. 5 and covers mainly the epidermis to obtain a quantitative measure for each evaluation procedure. Table 1 summarizes the result. Noticeable is the low fluctuation (∼3%) of the averaged intensity B-scans that indicates very stable OCT data acquisition, a prerequisite for dynamic OCT. While the reproducibility of the amplitude based dynamic OCT evaluation is very high (4-12%), both phase based evaluation procedures show a larger variation between the measurements (20-36%).

**Table 1.** Reproducibility data showing the mean and standard deviation of the region of interest (cf. Figure 5) for 4 successively recorded data sets.

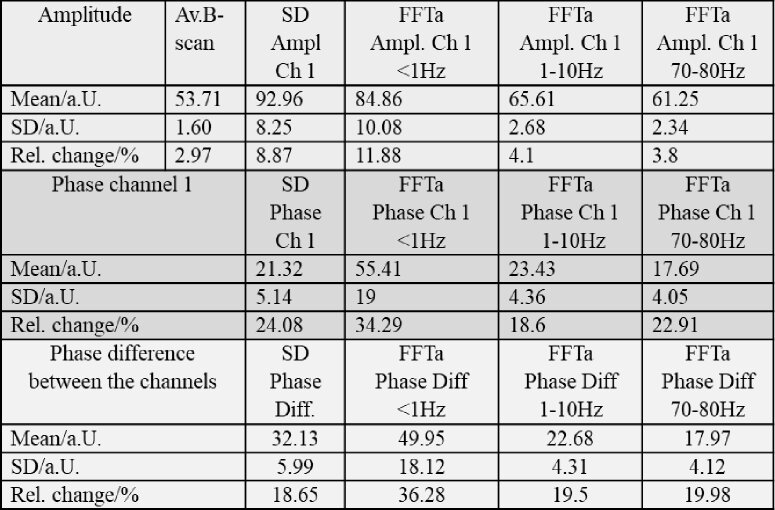

**Fig. 6.**
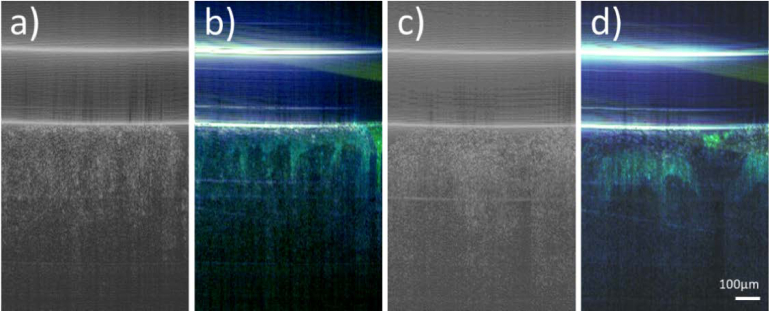
Representative images of living skin samples retrieved from two different subjects. a) and c) are the averaged intensity B-scans, b) and d) are false color images retrieved from a frequency analysis of amplitude fluctuations in channel 1. (Color scale: < 1 Hz: blue, 1-10 Hz green, 70-80 Hz: red). OCT data retrieved from a total of 512 B-scans.

To demonstrate the reliability of the method to detect cellular metabolism in the living epidermis, Fig. 6 shows other skin samples that have been retrieved from two other subjects yielding similar contrast from the living epidermis as in Fig. 3,

## 4. Discussion

We presented a novel dual beam OCT system that was used to investigate cellular dynamics in living and fixated human skin samples. Thereby, high cellular dynamics where observed mainly from the living epidermis (cf. Figures 3 and 5), a skin layer that is known to be very active due to a strong metabolism [32]. In fixated tissue we found no dynamics from this layer (cf. Figure 4). Interestingly, we found some dynamics, although in different frequency bands, from the stratum corneum in both the living and fixated sample. This layer consists only of cells without a nucleus and should not show cellular activity that is caused for example by motion of sub-cellular components or by cellular metabolism. However, the stratum corneum is capable of exchanging fluids that leads to swelling or shrinking of the cellular walls over time, potentially leading to the observed dynamic signals.

Our system allows to measure cellular dynamics simultaneously with two different lateral resolutions. As can be seen in Fig. 3, a lower lateral resolution results in a lower visibility of these dynamics. We hypothesize that at some point, with further decreasing lateral resolution, these cellular dynamics will not be observable at all. Therefore, the lateral resolution in respect to the cell size of tissue is an important aspect that needs to be considered for extracting dynamic OCT signals. In our configuration, we are certainly at the lower limit for skin tissue. Nevertheless, cellular structures are revealed in the dynamic images that are otherwise obscured by speckles in the standard OCT image (cf. Figure 3).

We want to point out here that our system differs from many other OCT systems that are used for skin imaging with respect to the imaging wavelength (1060 nm) and the transverse resolution (2.7-5 µm). As both factors strongly influence the intensity contrast, we observe a differing contrast in our images that might appear unfamiliar. Nevertheless, the lower intensity based contrast between the layers emphasizes the benefit of dynamic contrast that generates differences in contrast although hardly an intensity based contrast is visible.

We used a polarization sensitive beam splitter in the reference arm to clearly separate the light of both imaging beams while aligning them co-linearly. It must be noted, however, that polarization paddles are used for the fibers in the reference arm to match the polarization state exiting the fiber with the orientation of the polarizing beam splitter to maximize the optical throughput. Thus, after back propagation through the fiber in the reference arm, the light returning from both reference arms will be in a linear polarization state with the same orientation. We confirmed this experimentally by analyzing the polarization state of the light returning from the reference arm that exits the fiber at each 50:50 beam splitter that combines the light from sample and reference arm, respectively. A comparison of the intensity images of the two channels in Figs. 3 and 4 does not reveal any obvious polarization induced differences, further emphasizing that differences between the channels that are associated with polarization effects are small.

We tested different methods to extract cellular dynamics and we found that similar to previous studies [20] the FFTa yielded the best results. However, we additionally used solely the phase information to extract cellular dynamics (cf. Figure 3 g). Here, we want to point out that the amplitude and the phase of the OCT signal show similar temporal characteristics and therefore yield similar contrast in the images (cf. Figure 3 e, g). A slightly different contrast is obtained from the phase difference between the two OCT channels. Thereby, the image appears crisper than the image derived from the phase of channel 1 alone. The origin of this and potential correlations to anatomical structures are at the current stage unclear and need further investigations.

Dynamic OCT yields information on the metabolic activity of tissue. In our case we additionally used it to improve the image contrast in the images. To provide a quantitative assessment of the contrast enhancement we calculated the contrast to noise ratio (CNR) between regions of interest [33]. The regions of interest were manually selected and consist of squares each spanning 15 × 15 pixels and are retrieved from the stratum corneum, the living epidermis and the dermis, respectively. The CNR is defined in Eq. (1) [33] and the data is displayed in Table 2.

(1) $$CNR=\frac{\left|A_{region1}-A_{region2}\right|}{\sqrt{\sigma_{region1}^{2}+\sigma_{region1}^{2}}}$$

**Table 2.** Contrast to noise ratio (CNR) values between skin tissues for the different evaluation methods. str.c. stratum corneum, l.ep. living epidermis.

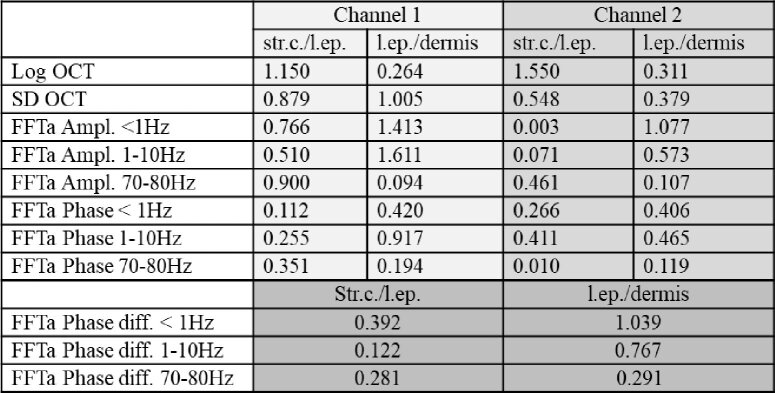

$A_{region1/2}$ indicates the average value within the region of interest, $\sigma_{region1/2}$ denotes the standard deviation of the values within the region of interest. The highest contrast between the living epidermis and dermis is observed by the FFTa analysis in the 1-10 Hz frequency range. Most of the contrast difference values between the two layers that are derived via dynamic OCT evaluation are higher for channel 1 in comparison to channel 2. Note that the intensity and dynamic images from the individual skin layers show internal structures that lead to the relatively low CNR values of the data.

The specific frequency bands were chosen after a FFT analysis for every pixel in the B-scan and subsequent averaging of the FFT profiles. This procedure enabled a determination of the dominating frequencies within the data. The frequency bands <1 Hz, 1-10 Hz and 70-80 Hz correspond well with an analysis provided by D. Nolte and potentially allows a separation between different metabolic activities or sub-cellular processes [34]. We want to emphasize here that pixels showing motion in the high frequency band were mainly observed from the stratum corneum while pixels in the other frequency bands are observed throughout the epidermis.

One pre-condition for the phase difference method to work is that the cellular dynamics are mainly visible in the higher resolution channel while the lower resolution channel serves as stable phase reference. The experimental realization of the dual beam setup leads to a relatively stable phase relation between the channels. However, we observed slow temporal drifts between the channels that are potentially caused by thermal drifts of the axial positions between the collimators of the two channels. Therefore, we used the phase difference value at the surface of the glass plate as reference which eliminates this influence.

**Fig. 7.**
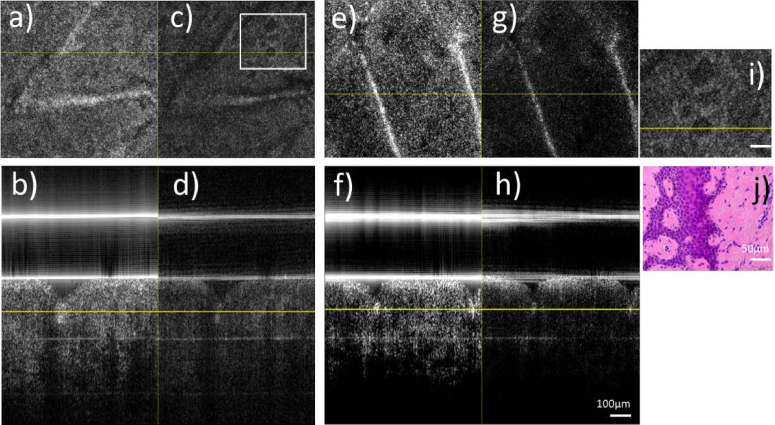
Images retrieved from 5 recorded 3D data sets using channel 1. a) and b) Averaged en-face intensity image and intensity B-scan of a living sample. c) and d) en-face dynamic and dynamic B-scan of a living sample. e) and f) Averaged en-face intensity image and intensity B-scan of a fixated sample, g) and h) en-face dynamic and dynamic B-scan of a fixated sample. i) is an enlargement of the region of interest marked by the white rectangle in c). j) En-face histologic slice retrieved from an adjacent region of the same skin region.

As can be seen in Fig. 5, the reproducibility of the method is very good. Averaging of several dynamic OCT images further improves the contrast and the signal to noise ratio of the images (cf. right column in Fig. 5). The broader applicability of the method is shown in Fig. 6 where the result of additional samples is shown. Although, our method is applicable for three dimensional imaging, the current measurement protocol would result in measurement times longer than 20 minutes (for 512 B-scans along the slow scanning axis) which seems not very practical, because during this time various factors such as temperature changes might influence the observed dynamics [16]. To test if a contrast improvement can be achieved within a shorter time frame, we recorded 5 volumes (each consisting of 512 × 512 A-scans) at the same location and calculated the average as well as the standard deviation of the amplitudes of the scan. The result is displayed for a living and fixated sample in Fig. 7. Similar as in the 2D imaging scenario using the amplitude variance, we observe increased contrast in the dynamic image of the living sample from the living epidermis layer, while in the fixated sample, dynamic changes are only observed in the stratum corneum.

During the review process of our work, two papers have been published that use phase based approaches for assessing cellular dynamics as well [35,36]. In the work of J.A. Bellesini et al. the complex OCT signal is exploited, i.e. the Fourier analysis is performed over the real and imaginary parts of the OCT signal. This approach mixes the amplitude and phase information of the OCT signal and leads to an improved contrast between breast tumor and surrounding tissue. Our approach is solely based on the phase information and yields similar images than the amplitude approach. However, differences between amplitude and phase based evaluation can be observed (cf. Figs. e) and g) and Tables 1, 2) that may provide additional information of the metabolism of the tissue. In the work of H. Schulz-Hildebrandt et al., the phase analysis is performed in another way as well. Instead of performing a FFT analysis over the recorded B-scans, the phase difference is calculated between successively recorded B-scans. The mean of the phase differences between all recorded B-scans as well as the standard deviation is exploited to generate a dynamic contrast. The frequency of the motion is thereby encoded in the measured phase difference and the set time difference between the B-scans.

A translation of our method to *in-vivo* imaging seems possible. However, a pre-requisite for this translation is the complete removal of motion that might be challenging. Although the glass plate provides some stability to the surface, varying pressure that is applied to the glass plate from the skin (introduced for example by breathing) will lead to a deformation of tissue in deeper layers and therefore obscure the dynamic signal. Thereby, the dual beam method might help to mitigate this issue as this method only requires a phase stability between the two channels and not over several B-scans.

## 5. Conclusion

We introduced a novel dual beam method for measuring cellular dynamics and metabolic activity in human skin samples. The living epidermis showed high activity and therefore increased contrast in the dynamic images that are otherwise not visible in the intensity images. The method has high potential to be applicable to *in-vivo* measurements and may represent an important tool for investigating changes in cellular activity and cellular metabolism that are caused by environmental influences.

## Data Availability

Data underlying the results presented in this paper are not publicly available at this time but may be obtained from the authors upon reasonable request.

## References

[1] Pils V. Ring N. Valdivieso K. et al., “Promises and challenges of senolytics in skin regeneration, pathology and ageing,” Mech. Ageing Dev. 200, 111588 (2021). 10.1016/j.mad.2021.111588 34678388

[2] Rajadhyaksha M. Gonzalez S. Zavislan J. M. et al., “In vivo confocal scanning laser microscopy of human skin II: Advances in instrumentation and comparison with histology,” J. Investig. Dermatol. 113(3), 293–303 (1999). 10.1046/j.1523-1747.1999.00690.x 10469324

[3] Branzan A. L. Landthaler M. Szeimies R. M., “In vivo confocal scanning laser microscopy in dermatology,” Lasers in Med. Sci. 22(2), 73–82 (2007). 10.1007/s10103-006-0416-8 17115235

[4] Koehler M. J. Konig K. Elsner P. et al., “In vivo assessment of human skin aging by multiphoton laser scanning tomography,” Opt. Lett. 31(19), 2879–2881 (2006). 10.1364/OL.31.002879 16969409

[5] Konig K. Riemann I., “High-resolution multiphoton tomography of human skin with subcellular spatial resolution and picosecond time resolution,” J. Biomed. Opt. 8(3), 432–439 (2003). 10.1117/1.1577349 12880349

[6] Ferrari M. Mottola L. Quaresima V., “Principles, techniques, and limitations of near infrared spectroscopy,” Can. J. Appl. Physiol. 29(4), 463–487 (2004). 10.1139/h04-031 15328595

[7] Welzel J., “Optical coherence tomography in dermatology: a review,” Skin Res. Technol. 7(1), 1–9 (2001). 10.1034/j.1600-0846.2001.007001001.x 11301634

[8] Wan B. Ganier C. Du-Harpur X. et al., “Applications and future directions for optical coherence tomography in dermatology,” Brit. J. Dermatol. 184(6), 1014–1022 (2021). 10.1111/bjd.19553 32974943

[9] Olsen J. Holmes J. Jemec G. B. E., “Advances in optical coherence tomography in dermatology-a review,” J. Biomed. Opt. 23(04), 1 (2018). 10.1117/1.JBO.23.4.040901 29701018

[10] Pircher M. Goetzinger E. Leitgeb R. et al., “Three dimensional polarization sensitive OCT of human skin in vivo,” Opt. Express 12(14), 3236–3244 (2004). 10.1364/OPEX.12.003236 19483847

[11] Dubois A. Levecq O. Azimani H. et al., “Line-field confocal optical coherence tomography for high-resolution noninvasive imaging of skin tumors,” J. Biomed. Opt. 23(10), 1 (2018). 10.1117/1.JBO.23.10.106007 30353716

[12] Israelsen N. M. Maria M. Mogensen M. et al., “The value of ultrahigh resolution OCT in dermatology - delineating the dermo-epidermal junction, capillaries in the dermal papillae and vellus hairs,” Biomed. Opt. Express 9(5), 2240–2250 (2018). 10.1364/BOE.9.002240 29760984 PMC5946785

[13] Ruini C. Schuh S. Sattler E. et al., “Line-field confocal optical coherence tomography-Practical applications in dermatology and comparison with established imaging methods,” Skin. Res. Technol. 27(3), 340–352 (2021). 10.1111/srt.12949 33085784

[14] Azzollini S. Monfort T. Thouvenin O. et al., “Dynamic optical coherence tomography for cell analysis [Invited],” Biomed. Opt. Express 14(7), 3362–3379 (2023). 10.1364/BOE.488929 37497511 PMC10368035

[15] Leroux C. E. Bertillot F. Thouvenin O. et al., “Intracellular dynamics measurements with full field optical coherence tomography suggest hindering effect of actomyosin contractility on organelle transport,” Biomed. Opt. Express 7(11), 4501–4513 (2016). 10.1364/BOE.7.004501 27895991 PMC5119591

[16] Apelian C. Harms F. Thouvenin O. et al., “Dynamic full field optical coherence tomography: subcellular metabolic contrast revealed in tissues by interferometric signals temporal analysis,” Biomed. Opt. Express 7(4), 1511–1524 (2016). 10.1364/BOE.7.001511 27446672 PMC4929658

[17] Monfort T. Azzollini S. Brogard J. et al., “Dynamic full-field optical coherence tomography module adapted to commercial microscopes allows longitudinal in vitro cell culture study,” Commun. Biol. 6(1), 992 (2023). 10.1038/s42003-023-05378-w 37770552 PMC10539404

[18] Scholler J. Mazlin V. Thouvenin O. et al., “Probing dynamic processes in the eye at multiple spatial and temporal scales with multimodal full field OCT,” Biomed. Opt. Express 10(2), 731–746 (2019). 10.1364/BOE.10.000731 30800511 PMC6377896

[19] Durand T. Paul-Gilloteaux P. Gora M. et al., “Visualizing enteric nervous system activity through dye-free dynamic full-field optical coherence tomography,” Commun. Biol. 6(1), 236 (2023). 10.1038/s42003-023-04593-9 36864093 PMC9981581

[20] Leung H. M. Wang M. L. Osman H. et al., “Imaging intracellular motion with dynamic micro-optical coherence tomography,” Biomed. Opt. Express 11(5), 2768–2778 (2020). 10.1364/BOE.390782 32499959 PMC7249806

[21] Abd El-Sadek I. Miyazawa A. Shen L. T. W. et al., “Optical coherence tomography- based tissue dynamics imaging for longitudinal and drug response evaluation of tumor spheroids,” Biomed. Opt. Express 11(11), 6231–6248 (2020). 10.1364/BOE.404336 33282486 PMC7687946

[22] Münter M. vom Endt M. Pieper M. et al., “Dynamic contrast in scanning microscopic OCT,” Opt. Lett. 45(17), 4766–4769 (2020). 10.1364/OL.396134 32870852

[23] Yang L. Ji P. Buzetta A. A. M. et al., “Longitudinal tracking of perfluorooctanoic acid exposure on mammary epithelial cell spheroids by dynamic optical coherence tomography,” Biomed. Opt. Express 15(9), 5115–5127 (2024). 10.1364/BOE.530775 39296396 PMC11407248

[24] Abd El-Sadek I. Morishita R. Mori T. et al., “Label-free visualization and quantification of the drug-type-dependent response of tumor spheroids by dynamic optical coherence tomography,” Sci. Rep. 14(1), 3366 (2024). 10.1038/s41598-024-53171-4 38336794 PMC10858208

[25] Münter M. Pieper M. Kohlfaerber T. et al., “Microscopic optical coherence tomography (mOCT) at 600 kHz for 4D volumetric imaging and dynamic contrast,” Biomed. Opt. Express 12(10), 6024–6039 (2021). 10.1364/BOE.425001 34745719 PMC8547980

[26] Mukherjee P. Fukuda S. Lukmanto D. et al., “Label-free metabolic imaging of non-alcoholic-fatty-liver-disease (NAFLD) liver by volumetric dynamic optical coherence tomography,” Biomed. Opt. Express 13(7), 4071–4086 (2022). 10.1364/BOE.461433 35991915 PMC9352293

[27] Leichtle A. Penxova Z. Kempin T. et al., “Dynamic Microscopic Optical Coherence Tomography as a New Diagnostic Tool for Otitis Media,” Photonics 10(6), 685 (2023). 10.3390/photonics10060685

[28] Ren C. Hao S. Y. Wang F. et al., “Dynamic contrast optical coherence tomography (DyC-OCT) for label-free live cell imaging,” Commun. Biol. 7(1), 278 (2024). 10.1038/s42003-024-05973-5 38448627 PMC10918170

[29] Dubois A. Levecq O. Azimani H. et al., “Line-field confocal time-domain optical coherence tomography with dynamic focusing,” Opt. Express 26(26), 33534–33542 (2018). 10.1364/OE.26.033534 30650800

[30] Wojtkowski M. Srinivasan V. J. Ko T. H. et al., “Ultrahigh-resolution, high-speed, Fourier domain optical coherence tomography and methods for dispersion compensation,” Opt. Express 12(11), 2404–2422 (2004). 10.1364/OPEX.12.002404 19475077

[31] Beer F. Patil R. P. Sinha-Roy A. et al., “Ultrahigh Resolution Polarization Sensitive Optical Coherence Tomography of the Human Cornea with Conical Scanning Pattern and Variable Dispersion Compensation,” Appl. Sci. 9(20), 4245 (2019). 10.3390/app9204245 31915537 PMC6949131

[32] Yamanishi H. Soma T. Kishimoto J. et al., “Marked Changes in Lamellar Granule and Trans-Golgi Network Structure Occur during Epidermal Keratinocyte Differentiation,” J. Investig. Dermatol. 139(2), 352–359 (2019). 10.1016/j.jid.2018.07.043 30240698

[33] Sanderson R. W. Fang Q. Curatolo A. et al., “Camera-based optical palpation,” Sci. Rep. 10(1), 15951 (2020). 10.1038/s41598-020-72603-5 32994500 PMC7524728

[34] Nolte D. D., “Coherent light scattering from cellular dynamics in living tissues,” Rep. Progr. Phys. 87(3), 036601 (2024). 10.1088/1361-6633/ad2229 38433567

[35] Bellesini J. A. Foo K. Y. Li J. Y. et al., “Three-dimensional dynamic optical coherence tomography for breast tumor margin assessment,” Biomed. Opt. Express 16(8), 3061–3074 (2025). 10.1364/BOE.563044 40809963 PMC12339312

[36] Schulz-Hildebrandt H. Gardecki J. A. Miller T. et al., “Phase-sensitive dynamic micro-optical coherence tomography for high-speed intracellular motion imaging,” Opt. Lett. 50(15), 4734–4737 (2025). 10.1364/OL.563024 40751978 PMC12801154

